# A comprehensive study for Al_2_O_3_ nanofluid cooling effect on the electrical and thermal properties of polycrystalline solar panels in outdoor conditions

**DOI:** 10.1007/s11356-023-25928-3

**Published:** 2023-02-21

**Authors:** Ali Ibrahim, Muhammad Raafat Ramadan, Abd EL-Monem Khallaf, Muhammad Abdulhamid

**Affiliations:** 1 Physics Department, Faculty of Science, Tanta University, Tanta, 31 527 Egypt; 2 Department of Basic Science, Misr Higher Institute for Engineering and Technology, Mansoura, Egypt

**Keywords:** Photovoltaic panels cooling, Al_2_O_3_ nanoparticles, Nanofluid cooling, PV efficiency enhancement

## Abstract

Photovoltaic (PV) technology is considered one of the most effective and promising renewable sources of energy. The PV system’s efficiency strongly depends on its operating temperature, which acts as a defect to the electrical efficiency by increasing over 25 °C. In this work, a comparison was performed between three traditional polycrystalline solar panels simultaneously at the same time and under the same weather conditions. The electrical and thermal performances of the photovoltaic thermal (PVT) system integrated with a serpentine coil configured sheet with a plate thermal absorber setup are evaluated using water and aluminum oxide nanofluid. For higher mass flow rates and nanoparticle concentrations, an improvement in the PV modules short-circuit current (*I*_*sc*_) and open-circuit voltage (*V*_*oc*_) yield and electrical conversion efficiency is achieved. The enhancement in the PVT electrical conversion efficiency is 15.5%. For 0.05% volume concentration of Al_2_O_3_ and flow rate of 0.07 kg/s, an enhancement of 22.83% of the temperature of PVT panels’ surface over the reference panel has been obtained. An uncooled PVT system reached a maximum panel temperature of 75.5 °C at noontime and obtained an average electrical efficiency of 12.156%. Water and nanofluid cooling reduce the panel temperature by 10.0 °C and 20.0 °C at noontime, respectively.

## Introduction

The demand for solar energy is increasing daily because of its huge availability and low cost; however, there is a huge problem related to the efficiency of energy conversion. Hence, to raise the ability of conversion, we have two ways: the first is through the early stage of the panel’s manufacturing and the other is by mechanical methods such as cleaning and cooling to reduce the losses that come out because of dust and overheating, respectively. The latter way is considered more economical than the first one which needs a lot of expensive facilities and is also still a point of research; however, this introduces an expensive/non-compatible model of solar panels. The conversion efficiency for traditional silicon solar panels lies in the range of 15–18% (Tatsuo Saga 2010; Sargunanathan et al. 2016). Semiconductors need to be heated to conduct the current until a specific temperature value depends on the type of semiconductor. By exceeding its temperature, the heating is involved as a degradation agent towards the output. For mono- and poly-crystalline silicon solar panels, when they get overheated at temperatures above 25 °C, their efficiency drops by 0.5% as the temperature increases by 1 °C (Suresh et al. 2018).

There are many types of solar panels such as mono- and polycrystalline silicon solar cells, thin films, and organic solar cells. The main difference between mono- and polycrystalline solar cells is the manufacturing way, efficiency, and economic cost. Polycrystalline silicon solar panels have randomly oriented boundaries of their grains which make them less efficient than monocrystalline panels which have mostly organized grains (Seager 1985, Karki 2015).

Charge carriers (i.e., electrons and holes) in the semiconductors are generated due to the absorption of the penetrating photons with energy exceeding the bandgap energy. The increment of solar irradiance and temperature have two contrary effects on the solar panel’s voltage and current. As the irradiance increases, the values of *V* and *I* increase as well. In contrast, as the temperature increases, the value of *V* drops, while the value of *I* slightly increases. The electrical characteristics of the semiconductor originally deformed due to the mismatch of lattices, which results from the non-similar orientation of grains (Seager 1985). In addition, the charge carriers’ recombination introduces a heating effect (Karki 2015). Hence, the polycrystalline solar panel has fewer electrical properties than mono-crystalline silicon solar panels. For a polycrystalline solar panel, as a semiconductor, the temperature effect has an advantage as the temperature is raised to a specific value and then it will have a negative effect. A study is done to compare the efficiency of a monocrystalline silicon panel at temperatures of 25 and 60 °C, and it is found to be 13.3 and 10.3%, respectively (Radziemska, E. 2003a).

A promising solution for this issue of overheating is to cool down the solar panels to enhance their efficiency. The reduction of the solar panel’s temperature which affects the ohmic resistance directly and conversely increases the values of current, power, and total efficiency. Many ways are used to cool the panel such as air cooling which is used to compare two panels with and without back channels (Mazon-Hernandez et al. 2013) and water spraying on the module’s surfaces (Moharram et al. 2013; Nizetic et al. 2016) that induces more cooling due to its higher ability for heat transfer than air. Open-circuit water cooling has been carried out using three ways, i.e., from the upper side only that has a demerit of increasing the reflectivity of the incident solar radiation (Abdolzadeh and Ameri 2009), from the downside only (Bahaidarah et al. 2013), or both at the same time (Nizetic et al. 2016) showing an increase in the mean efficiency by 3.26%, 9%, and 14.1%, respectively. Examining the water spray cooling effect on the efficiency in indoor conditions using a sun simulator with different values of irradiance has shown an improvement in the power by 9 to 22% due to the reduction in the temperature by 5–22 °C (Irwan et al. 2015). The impact of temperature on the performance of PV and PV thermal (PVT) systems has been studied by many researchers (Chow 2010, Radziemska, E. 2003a, Sacco et al. 2013, Meneses-odrgue et al. 2005, Orioli and Gangi 2013, Zaoui et al. 2015, Vittorini et al. 2017, Al-Addous et al. 2017, Sajjad et al. 2019, Al-rwashdeh 2018, Ammar et al. 2019a, Taner 2018, Taner 2015, Taner 2017). Chander et al. (2015) experimentally investigated the effect of temperature on the behavior of the mono-crystalline solar cell. The experiments were carried out at 550 W/m^2^ light intensity and a temperature of the solar panel of 25–60 °C. Cuce et al. (2013) investigated experimentally the effect of light intensity and temperature on the performance of the PV panel. The output power of the solar cell can be decreased by about 0.4% with an increase in its temperature of 1 K (Radziemska, E. 2003b). The performance of the PVT system can be improved by changing the types and consequently thermo-physical properties of base fluid (Ibrahim et al. 2009).

Since, the obtained results explained (Jaisankar et al. 2009) that the nanoparticles are the best solution for improving the heat transfer characteristic of the PVT system (Sani et al. 2010, Wong and Leon 2010, Sardarabadi et al. 2017a, Al-Waeli et al. 2017, Hasan et al. 2017, Al-Shamani et al. 2015). Some recent studies on using nanofluids are reported (Ammar et al. 2019b, Ghadiri et al. 2015, Michael and Iniyan 2015, Sardarabadi et al. 2017b, Hassani et al 2016, Sardarabadi et al. 2014, Guo et al. 2015, Mebarek-Oudina et al. 2018, Alkasassbeh et al. 2019, Mebarek-Oudina 2017, Mebarek-Oudina 2019, Abbas et al. 2019).

Nanofluid is a term that refers to nanoparticles that are suspended in a base fluid such as water, ethylene glycol, and oil. Cooling the solar panels through fluids is a promising technique due to the thermal contact between the nanofluid molecules and the body of the panel, which facilitates a heat-transferring process to take a place. However, the heat capacity of the base fluid plays an important role in the cooling process. Hence, nanofluid is more efficient than the single base fluid as a cooler. Nanofluid cooling is a more reliable technique than water cooling due to its higher heat capacity and thermal conductivity. Different studies have been conducted to check the ability to use nanofluids to cool solar panels. Cooling the photovoltaic cells with nanofluids with different concentrations and base fluid such as ethylene glycol has shown a better enhancement than water as a base fluid (Esfe et al. 2014). An efficiency enhancement of 33.27% when using an aluminum box of 3-mm thickness and SiC nanoparticles with water-based fluid at a concentration of 0.5% and a flow rate of 2 l/min as reported by Abbood et al. (2020). A comparative study is made between Al_2_O_3_, CuO, and Al_2_O_3_-CuO mixture nanofluids (Shankar Amalraj et al. 2019). The obtained results of this study have shown a better efficiency for cooling through Al_2_O_3_-CuO, CuO, and Al_2_O_3_, respectively (Shankar Amalraj et al. 2019). Investigation of Al_2_O_3_ nanofluid with water-based cooling compared to T_i_O_2_ at 0.1% has shown better results as reported by (Ebaid et al. 2020). On the other hand, another study has been done using the same two nanoparticles with base-fluid mixtures, water-cetyltrimethylammonium bromide for Al_2_O_3_, and water-polyethylene glycol for T_i_O_2_ at different concentrations and flow rates. It is found that the first case has better performance than the second (Ebaid et al. 2018). Most of the studies have been undertaken in conventional PVT design systems that added an absorber plate at the back of the PV panel. In addition, cross-sectional areas of the cooling channel were often circular or rectangular. Since the rising temperature of the PV cells leads to decreasing electrical efficiency, a design with a more efficient cooling method could increase both electrical and thermal efficiency. A study performed by (Abdo and Saidani-Scott 2021) used alumina-saturated with hydrogels at concentrations of 0.1%, 0.25%, and 0.5% in comparison to water-saturated with hydrogel cooling and no-cooling at solar irradiance range 800–1000 W/m^2^ to get the best economic and environmental gain.

The objective of our study is to examine the ability to use a new geometry of a heat exchanger at the back of the module instead of spraying the water or nanofluid over the surface of the panel. This causes a loss and evaporation of water used for the cooling process at the time of its tremendous need. In addition, nanofluids are high-cost and cannot be used, unless in a closed cooling circuit. To achieve the set objective, a solar-thermal collector is attached to the back of the PV modules to absorb the waste heat from the modules. Also, both the electrical performance and thermal properties of the PVT will be carried out.

## Experimental setup

**Fig. 1:**
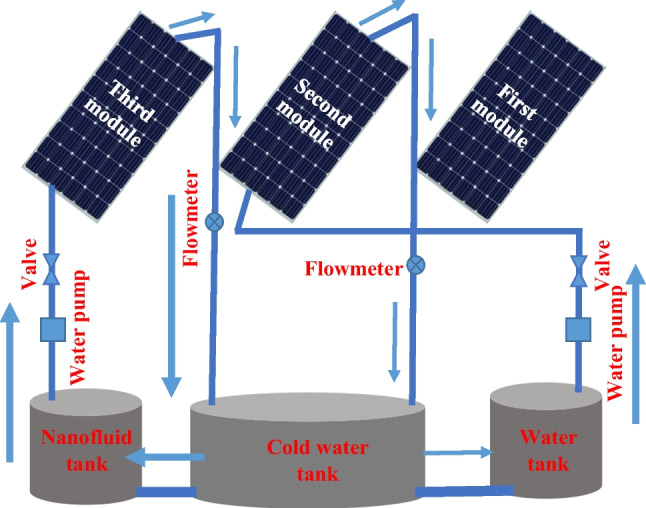
Schematic diagram of the PVT system

A schematic diagram of the PVT with a cooling process system is shown in Fig. 1. By cooling the PV module, electrical performance can be improved. Where, the numbers 1, 2, and 3 represent the three panels: reference, cooled by water, and cooled by Al_2_O_3_ nanofluid, respectively. Starting with the pump, which pulls the fluid from the tanks and pushes it through the pipes under the panel. Consequently, the fluid gets out to the hot fluid exchanger, to get cooled. Finally, the outlet fluid returned to the tanks again to be recycled. Flowmeters 1 and 2 are used to measure the flow rates of the water and nanofluid, respectively. The valves are used to control the flow rate at the desired values. Two sensors are used: to measure the temperatures of the inlet and outlet fluids. At the same time, the surface temperature of each panel is recorded through another three temperature sensors on the surface, besides the ambient and the hot fluid exchanger temperature.

**Fig. 2:**
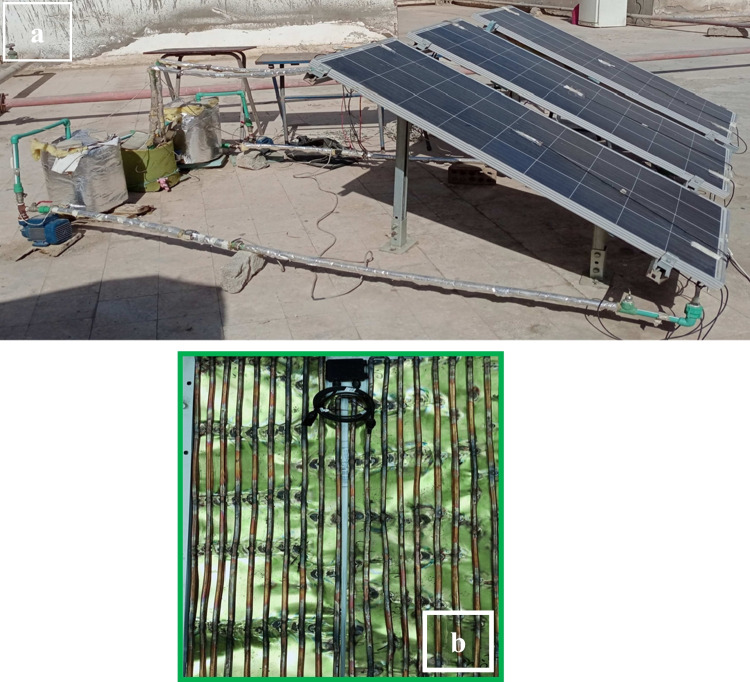
Photograph of assembly of various components of a PVT system. (**a**) Whole PVT system. (**b**) Serpentine heat exchanger at the back of the solar panel

A photograph of the actual system used in the experimental work contains PV polycrystalline solar panels, pumps, tanks, temperature sensors, and transferring pipes, as shown in Fig. 2(a) and (b). A heat exchanger, as depicted in Fig. 2(b), is composed of semi-rectangular tubes of copper with a diameter of 0.93 cm; each pipe has a length of 145 cm and is welded to a sheet of copper approximately equal to the inner area of the panel. The benefit of this sheet is covering the whole panel’s area; thus, the copper sheet is cooled firstly through pipes, then the back of the module is cooled, consequently. The separation between each pipe is 2 cm. After installation of the pipes and attaching them to the copper sheet, the heat exchanger (i.e., the copper pipes and the copper sheet) is held in the frame by wood arms equal to the inner width (64 cm) of the panel and separated by 15 cm. Finally, the panel’s back is covered well by an isolating layer to prevent any other effects (i.e., only considering the fluids cooling).

### System components

#### Polycrystalline PV solar panels

Three identical solar panels of polycrystalline type from Power Field Company, Egypt are used. Table 1 shows the characteristics of polycrystalline PV panels, while theses specifications are measured under the temperature condition of 25 °C and at *E*=1000 W/m^2^.

**Table 1:** Solar PV panel characteristics

Parameter	Symbol	Value	Unit
Rated maximum power	*P*_*max*_	155.3	watt
Current at *P*_*max*_	*I*_*mp*_	8.263	Amp
Voltage at *P*_*max*_	*V*_*mp*_	18.14	volt
Short circuit current	*I*_*sc*_	8.39	Amp
Open-circuit voltage	*V*_*oc*_	22	volt
Conversion efficiency	*Η*	14.88	%
Weight	*W*	11.5	Kg
Nominal operating temperature	*T*_*n*_	47 ± 2	°C

#### Cold fluid heat exchanger

**Fig. 3:**
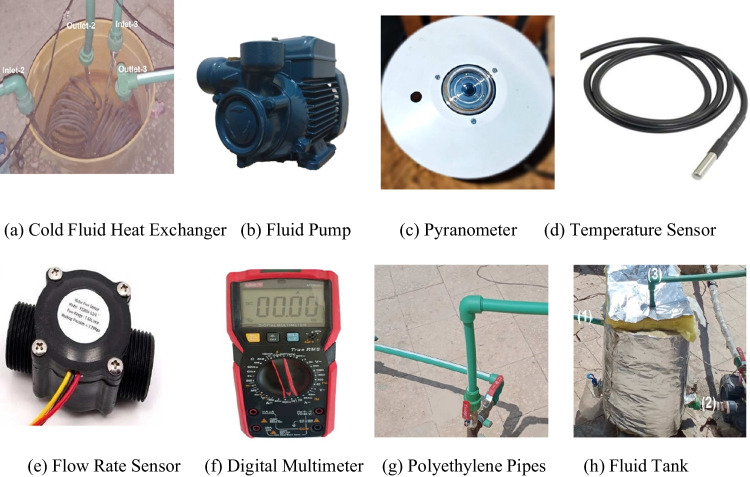
Tools of the experimental work

This part in Fig. 3a is used to reduce the temperature of the output fluid before it is returned to the tank again. The temperature of the cold water is approximately adjusted as $\approx 27-$ 30 °C. The pipes are converted to a helical-shaped copper tube, to increase conductivity between transferred fluid and cold water in this exchanger.

#### Fluid pumps

The fluids are forced through two pumps of (Pedrollo Corded Electric (PKm60)) type as in Fig. 3(b). The pump with a power of 0.5 horsepower with a volume flow rate reaching 90 L/m and a length of 100 m. The liquid temperature must be at a range of −10 to +90 °C for better usage.

#### Pyranometer, temperature sensors, flowmeters, and digital multimeter

**Fig. 4:**
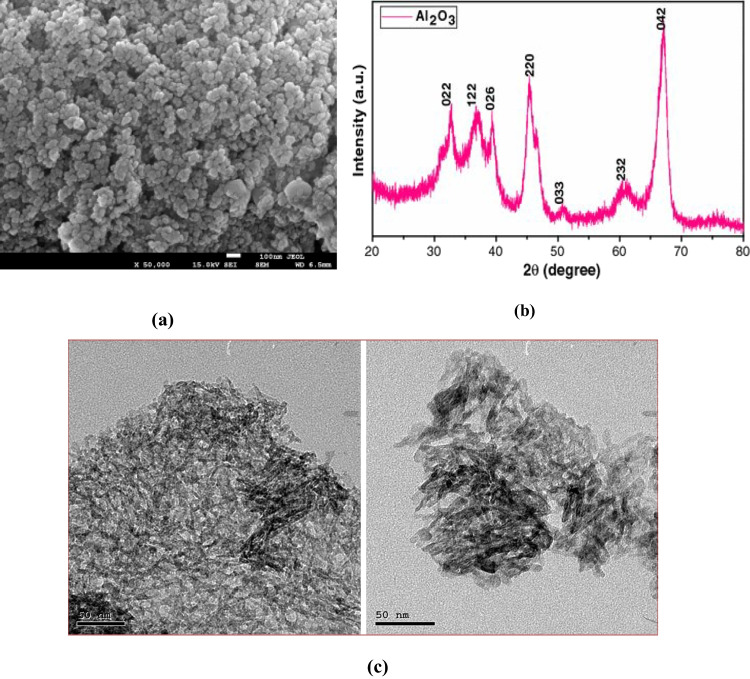
(**a**) Scanning electron microscope of Al_2_O_3_. (**b**) XRD pattern of Al_2_O_3_ nanoparticles. (**c**) TEM pattern of Al_2_O_3_ nanoparticles

For solar radiation measurements, a pyranometer (Eppely Radiometer) as shown in Fig. 4(c) was used. Since the site location is the roof of the Faculty of Science, Tanta City (${29.25}^{^\circ }$) latitude angle, Egypt. The waterproof temperature sensor in Fig. 3(d) of type (DS18B20) with a range lies between − 55 and + 125 °C with an accuracy of 0.5 °C. The distribution of temperature sensors was the following: firstly, nine temperature sensors were at the top of the panels (three for each one) and we calculated the average for each panel’s temperature. Secondly, four temperature sensors in the back of the two modules are cooled by water and nanofluid. Thirdly, four temperature sensors (two for the inlet and the outlet for each) for the two modules cooled by water and nanofluid. Finally, one temperature sensor is used for measuring the ambient temperature The flow sensor of the (FS300A G3/4) type in Fig. 4(e) is used to measure the mass flow rate of the fluid. The (UT89X) digital multi-meter as pictured in Fig. 4(f) is used to measure the values of voltage and current obtained from each panel. Both, heat, and flow rate sensors are depending on an Arduino circuit to convert the electrical signal obtained from these sensors into digital numbers which are presented on the personal computer attached to the system to register their readings every minute.

#### Polyethylene pipes and tanks

For the fluid transfer into tanks, shown in Fig. 3(g–h), polyethylene pipes of 1.25-cm diameter are used. The first pipe, no. (1) is the outlet fluid that is coming from the serpentine and passing through the hot-fluid heat exchanger; on the other hand, no. (2) is the inlet of fluid pulled from the tank to enter the serpentine, no. (3) is the passage of auxiliary (excess) fluid due to specifying the flow rate amount. To reduce the thermal contact with the ambient, they are well-isolated using glass wool, with an outer reflective surface to reflect the incident radiation.

#### Measuring techniques

The measuring process is divided into two stages: The first is measuring the short-circuit current (*I*_*sc*_) and the open-circuit voltage (*V*_*oc*_) for each panel every minute by using only the digital multimeters, connected directly with the output of the panel without any external load resistance. The second one is the current-voltage (IV) curve characterization. Firstly, we specify the *I*_*sc*_ value by just the digital multimeters, then measuring *I* and *V* values for each panel after adding a load resistance to each circuit. Current and voltage output from panels measured simultaneously with a gradual increase in the load resistances. The load resistance initially is a nickel chrome wire (1–10 ) to allow the identification of data points at higher values of output current, otherwise the small-valued resistances will be burned. Then, a variable resistor (a box of fixed resistances) is facilitated to obtain *I* and *V* at higher ranges of resistances. The most observed values of the load resistance to have *I*_*sc*_=0 and *V*_*oc*_ is at its maximum ranging from 1200 to 1500 . At the time of measuring the electrical output of each panel manually, the temperature behavior is recorded for each in a computerized way every minute.

#### Methodology

Using three polycrystalline PV solar panels operate under the same weather conditions, the first panel is considered as a reference, i.e., without any cooling technique. On the other hand, the second and third ones cooled through water and Al_2_O_3_ nanofluid, respectively. To get the best performance, different values of the concentrations for the nanofluid of 0.01%, 0.03%, and 0.05% are used at different values of mass flow rates of 0.01, 0.03, 0.05, and 0.07 kg/s are studied in a single day, reaching 12 days to cover all cases. The priority is for the highest concentration of 0.05%. Afterward, this nanofluid is diluted to obtain a lower concentration of 0.03%, and so on. After diluting, the sonication step takes place again.

#### Pre-adjustments

Firstly, the different values of mass flow rates are investigated for the same nanofluid concentration on four sequential days. Then the concentration of the nanofluid is changed to another with the same mass flow rate. The first and second valves are adjusted to get the same desired value of mass flow rate for the two modules. Then temperature sensors are checked, and multimeters are prepared for the measurement.

## Al_2_O_3_ Nanofluid preparation and characterization

Nanofluid is composed of nanoparticles and the base fluid, which makes the base fluid gain more thermal conduction properties (Ghadimi et al. 2011, Yu and Xie 2012, Devendiran and Amirtham 2016, Chamsa-Ard et al. 2017, Naser Ali et al. 2018, Ibna Ali et al. 2020). These nanoparticles may be metals such as Cu, Al, and Fe or metal oxides such as CuO, Al_2_O_3_, TiO_2_, and Fe_2_O_3_. Two methods are used for nanofluid synthesis, i.e., one-step and two-step methods, which is the most economic and easier one (Yu and Xie 2012). In this method, initially, the Al_2_O_3_ nanopowder is prepared and then dispersed into the base fluid (distillated water) with the help of intense magnetic force agitation. After that, for increasing the suspension of nanoparticles and the nanofluid stability against agglomeration, an emulsion of both nanoparticles and the base fluid by using a sonicator has been obtained.

**Fig. 5:**
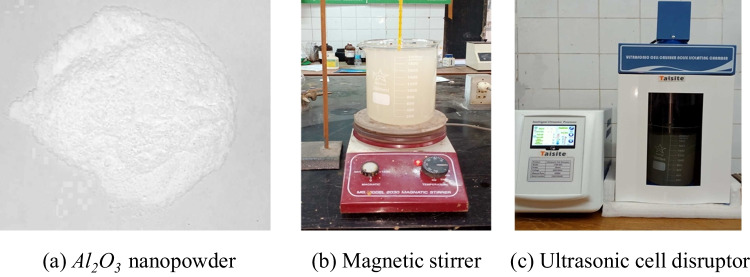
Preparation tools of nanofluid

Aluminum-metal oxide (Al_2_O_3_) is regarded as one of the most used nanoparticles to manufacture an effective nanofluid due to its high thermal conductivity (40.0 W/m K) (Teng and Hung 2014; Tanakaet al. 2001; Korsonet al. 1969). The white-colored alumina in Fig. 5(a) was as prepared nano-aluminum oxide by Nanogate Company, Cairo, Egypt. The average particle’s size is less than 30 nm with a spherical-like intact shape in Fig. 4(a). The XRD and TEM investigations are presented in Fig. 4(b–c).

### Tools and preparation method

#### Tools

There are many tools used for preparing the nanofluid: (i) Sensitive balance; the balance is used as the first step of preparation to determine the mass of nanopowder, shown in Fig. 5(a). (ii) Heater and magnetic stirrer; for 1 h, the 2-l sample is stirred at 40 °C and heated up to 70 °C by using MG Model 2030 type magnetic stirrer shown in Fig. 5(b). (iii) Sonicator and ultrasonic cell disruptor; after making the mixture using the stirrer, the sonication process is used for increasing the suspension of nanoparticles and reducing the agglomeration. The sonication process time is about 2 h. (iv) Ultrasonic cell disruptor (JY99-IIDN) shown in Fig. 5(c) has been used. A probe (*φ*25 type) is used for a sample range of 500–2000 ml and a power rate of range 30–95%. In this work, for the 2000 ml sample, an 80% power rate is used.

### Preparation method

The volumetric concentration and the mass of the nanoparticles can be calculated by equation (1) (Hussein et al. 2013), in the case of given *φ*%.

1 $$\mathrm{\varnothing }\%=\frac{({M}_{np}/{\rho }_{np})}{\left({M}_{np}/{\rho }_{np}\right)+({M}_{nf}/{\rho }_{nf})}$$

Then add the sample of nanopowder 3.88 gm to 2 l of the distilled water and stir with heat at 80 °C for 1 h. Finally, the mixture is sonicated in the ultrasonic sonicator for 1.5 h to reduce the possibility of agglomeration, where *M*_*np*_, *ρ*_*p*_, *M*_*nf*_, and *ρ*_*nf*_ are the mass and density of nanopowder and nanofluid, respectively. However, our system is an active one, meaning that there is less probability for nanoparticles to hold together unless the system does not work for 2 weeks. In this case, the nanoparticles will precipitate due to their effect of gravitational force in comparison to the viscous force.

Figure 4 (a) shows the XRD of the Al_2_O_3_ nanoparticles. It was found that the diffraction peaks $2\theta$ ~ 33.0°, 37.5°, 39.5°, 46.0°, 51.5°, 61.0°, 67.7°, and 66.5° have appeared. It is referred to $<022>$, $<122>$, $<026>$, $<220>$, $<033>$, $<232>$, and $<042>$ favorite directions of Miller indices respectively. Moreover, the results showed that the nanoparticles have a hexagonal structure. Figure 4 b shows the SEM morphology structure of the Al_2_O_3_ nanoparticles. They are characterized by their crystalline shapes with homogeneous sizes and spherical and semi-spherical shapes. Also, it shows that Al_2_O_3_ particle size distribution ranges from 25 to 40 nm, and the average was around 32.5 nm. These results agree with the results of XRD. Finally, Fig. 4c shows TEM images of the Al_2_O_3_ nanoparticles, which illustrated the presence of hexagonal nanoparticles $<50- \mathrm{nm}$ particle size.

**Fig. 6:**
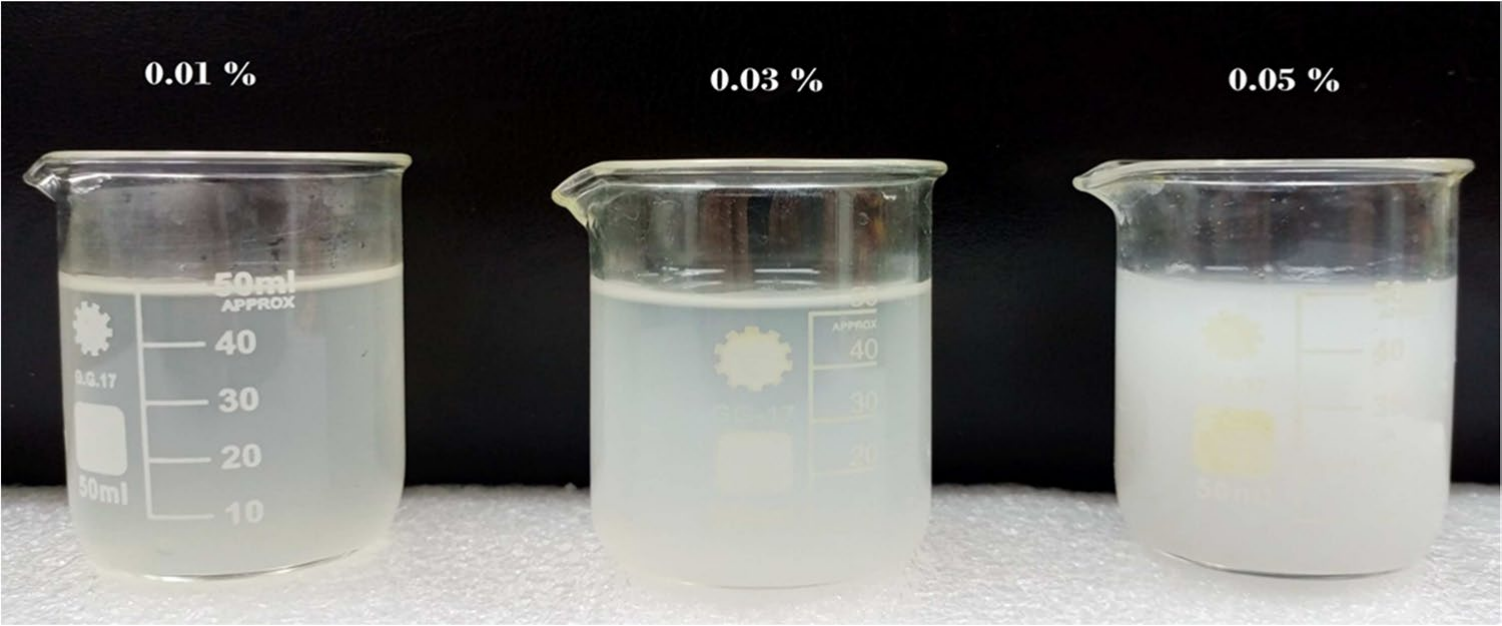
Overview of prepared Al_2_O_3_ nanofluid at different concentrations

A photograph of the prepared Al_2_O_3_ nanofluid at different concentrations is shown in Fig. 6.

### Thermophysical properties of the nanofluid

The thermophysical properties, i.e., PH, volume concentration, density, viscosity, specific heat, and thermal conductivity of the nanofluid could be measured or calculated theoretically through a set of equations built due to modeling of previous experimental measurements. The density (*ρ*_*nf*_) of the nanofluid is calculated by Eq. (2) (Pak and Cho^1998^; Drew and Passman 1999; Geliset al. 2022).

2 $${\rho }_{nf=}{\rho }_{bf}\left(1-\mathrm{\varnothing }\right)+{\rho }_{nf}\mathrm{\varnothing }$$

Equations (3) (Ibna Ali et al. 2020, Safieiet al. 2020) and (4) (Ibna Ali et al. 2020, Brinkman 1952) are used for calculating the dynamic viscosity (*μ*_*nf*_), for low volume concentrations in the range of (*ϕ* = 0*.*01%), and higher values of *ϕ*% limited to 4%, respectively.

3 $${\mu }_{nf}=(1+2.5\varnothing ){\mu }_{bf}$$

4 $${\mu }_{nf}=\left[\frac{1}{{\left(1-\mathrm{\varnothing }\right)}^{2.5}}\right]{\mu }_{bf}$$

The specific heat (*C*_*nf*_) and the thermal conductivity (*K*_*nf*_) of the nanofluid are calculated by Eqs. (5) (Popa et al. 2017, Zhou and Ni 2008) and (6) (Ibna Ali et al. 2020, Amin et al. 2021), respectively.

5 $${C}_{nf}={C}_{bf}\left(1-\mathrm{\varnothing }\right)+{C}_{np}\varnothing$$

6 $${K}_{nf}=\left[\frac{{K}_{np}+2{K}_{bf}+2\mathrm{\varnothing }\left({K}_{np}-{K}_{bf}\right)}{{K}_{np}+2{K}_{bf}-\mathrm{\varnothing }\left({K}_{np}-{K}_{bf}\right)}\right]{K}_{bf}$$

The values of the thermophysical properties, i.e., density, dynamic viscosity, specific heat, and thermal conductivity of water and Al_2_O_3_ nanofluid are listed in Table 2 and as a function of nanofluid in Table 3.

**Table 2:** Thermophysical properties of the Al_2_O_3_ nanofluid

	Water	Al_2_O_3_ nanofluid
Density (Kg/m^3^) at 25 °C	997.0470 (Tanaka et al 2001)	3880 (Teng and Hung 2014)
Dynamic viscosity (mPa.s) at 25 °C		0.8903 (Korson et al. 1969)
Specific heat (J/Kg °C)	4179 (Popa et al. 2017; Incropera et al. 1996)	779.2195 (Popa et al. 2017, Incropera et al. 1996, Lide David and Frederikse 1978)
Thermal conductivity (W/m K) at 27 °C	0.6096 (Ramires et al. 1995)	40.0 (Lide David and Frederikse 1978)

**Table 3:** Thermophysical properties as a function of nanofluid concentrations

ϕ $\%$	Density $(\mathrm{Kg}/{\mathrm{m}}^{3})$	Dynamic viscosity (mPa.S)	Specific heat (J/$\mathrm{K}$ g K)	Thermal conductivity (W/m K)	PH
0.01%	1000.0288	0.89032	4178.9660	0.609617	7.6
0.03%	1000.0864	0.89036	4178.8980	0.609652	7.4
0.05%	1000.1440	0.89041	4178.8300	0.609687	7.3

It is clear from Table 2 that the calculated values of the density, dynamic viscosity, and thermal conductivity of the nanofluid slightly increase with the nanoparticle’s concentration. On the other hand, both specific heat and PH (at 25 °C) values of the nanofluid are slightly decreased with the nanoparticle’s concentration.

### Stability

Any change in the fluid shape, particle distribution, suspension, agglomeration, or precipitation will negatively affect the ability of enhancement. So, the stability of the nanofluid is the most effective factor for the nanofluids. In our system, according to the active circulation process of the fluid, there is a daily re-mixture for the nanoparticles and the base fluid. However, keeping the system static for 1 week or more may require another sonication cycle for the fluid sample.

### Fluid flow and Reynold’s number

From dimensionless Reynold’s equation (Reynolds 1883; Ryan and Johnson 1959):

7 $$\mathrm{Re}=\frac{\rho vD}{\mu }$$

The fluid flow type whether it is turbulent or laminar is determined. If the Re exceeds 2100, it will be a turbulence flow (Reynolds 1883; Trinh, K. T. 2010). In Reynold’s equation, $\rho$ is the density of the fluid (Kg/${\mathrm{m}}^{3}$), $v$ is the flow velocity (m/s), $D$ is the diameter of the pipe (m), and $\mu$ is the dynamic viscosity (Pa.s). From Eq. (11), the values of the Reynold for the two flow rates: 0.03 and 0.07 kg/s at different volumetric concentrations 0.01%, 0.03%, and 0.05% are given in Table (4), depending on dynamic viscosity and density of the alumina nanofluid from Table (3).

**Table 4:** Reynold at different concentrations and flow rates

Volumetric concentration ($\varnothing$%)	Flow rates (Kg/s)	
	0.03 (Kg/s)	0.07 (Kg/s)
0.01%	4612.9462	10,762.4965
0.03%	4614.0046	10,762.6329
0.05%	4613.0631	10,762.6484

From the previous table, we can conclude that the flow of the fluid at different concentrations and flow rates is turbulent.

## Characterization of power and efficiency

The maximum power (*P*_*m*_) can be determined graphically through the IV-curve of the solar panel, which drawn by the variation of the load resistance. Then we can get graphically the maximum current *I*_*m*_ and the maximum voltage *V*_*m*_. Hence, *P*_*m*_ is given by (Goetzberger et al. 1998):

8 $${P}_{m}={I}_{m}\times {V}_{m}$$

The ability of a solar panel to convert absorbed solar radiation to electrical energy or the efficiency of the energy conversion (*η*) is given as a function of solar irradiance (*E*) in W/m^2^ and the effective area of the solar panel (*A*_*S*_) in m^2^ using the following equation (Goetzberger et al. 1998):

9 $$\eta=\frac{\mathrm{output}\;\mathrm{power}}{\mathrm{input}\;\mathrm{power}}=\frac{P_m}{E\times A_s}$$

The fill factor is a measure of the whole performance of the solar panel. The higher the fill factor, the higher power is produced (Karki 2015). It is given as a function of the open-circuit voltage (*V*_*oc*_*)* i.e., at zero load resistance, short-circuit current (*I*_*sc*_) (at high value of load resistance reaching to 1500 Ω at maximum, in our case), and maximum power (*P*_*m*_) by the following relation (Goetzberger et al. 1998):

10 $$\mathrm{FF}=\frac{{P}_{m}}{{V}_{oc}{I}_{sc}}$$

## Results and discussion

The testing of efficiency improvement has been carried out in the Faculty of Science, Tanta University, Tanta, Egypt in August, and September 2021. The typical measuring time is during the 12:00 – 2:00 P.M. period (i.e., the highest value of solar radiation). The highest measured solar irradiance during this period is 1200 W/m^2^, and the average value of the ambient temperature has been measured; it depends on the time and the day of measurement (the data not included). The surface temperatures of the three modules are recorded instantly using three temperature sensors for each; in addition, the inlet and outlet temperatures of the cooling fluids were recorded to check the effect of the hot panels on changing the fluids’ temperature. There are two graphs/every day representing the effect of temperature decrease on the power—measured every minute for about 10–15 min by starting the cooling process and the overall efficiency of each panel measured through the IV characteristic curve. The output of the solar panel is also affected by the mean solar irradiance of the day. In all the power/minute measurement graphs, there is a fluctuation in the values of power because of the disturbance of solar irradiance. Higher concentrations and higher mass flow rates could be the reason for better enhancement. In addition, the temperature characterization for each panel is added, represented by measuring the average surface temperature. It is found that for fluid cooling cases, the value of voltage is equal as the load resistance is increased; inversely, the current for the three panels is found to be approximately equal as the voltage is zero.

In this work, we started with the higher mass flow rates that are for different concentrations. While by increasing the nanofluid concentration leads to enhancement of the thermal conductivity that raises that heat transfer rate. To calculate the enhancement efficiency for the two other modules in comparison to the reference module, we use Eq. (11). In general, the results have shown that as the concentration and the flow of the nanofluid rate are increased, the improvement in the efficiency will be getting higher. Exceptionally, some results do not agree with the latter statement. This may be due to various weather conditions, whereas all study cases of our experimental work have proved that nanofluid cooling is better than water cooling.

11 $$\eta \%=\frac{{\eta }_{\mathrm{fluid} }-{\eta }_{\mathrm{ref}.}}{{\eta }_{\mathrm{ref}.}}$$

### With fluid concentration = 0.05%

**Fig. 7:**
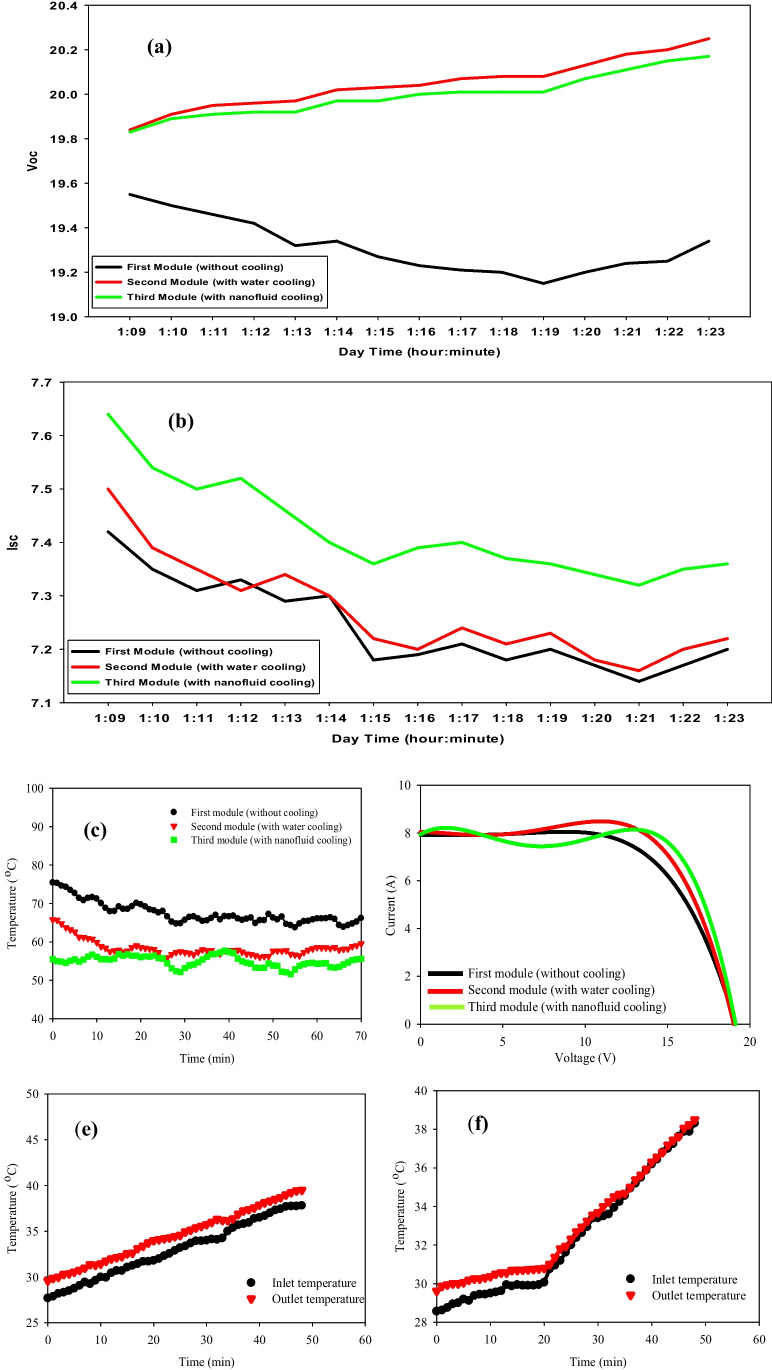
PV modules’ electrical characteristics and temperatures due to mass flow rate of 0.03 kg/s and concentration of 0.05% of nanoparticles. (01–09-2021). (**a**) Short-circuit current vs. time (1:09 P.M. to 1:23 P.M.). (**b**) Open-circuit voltage vs. time (1:09 P.M. to 1:23 P.M.). (**c**) Current–voltage characteristics of the three PV. (**d**) Surface module temperature vs. time (1:09 P.M. to 2:05 P.M.). (**e**) Second PV module inlet and outlet temperatures vs. time (1:08 P.M. to 2:05 P.M.). (**f**) Third PV module inlet and outlet temperatures vs. time (1:08 P.M. to 2:05 P.M.)

On September 1, 2021, the hourly variations in solar radiation and ambient temperature from 1:00 P.M. to 2:00 P.M. during the experimental period have been measured, where average solar radiation is 861.5 W/m^2^. In addition, the average ambient temperature is 38.5 °C. It is clear from the data that ambient air temperature is directly proportional to solar radiation. A maximum solar radiation intensity of 861.5 W/m^2^ and ambient air temperature of 38.5 °C is observed during the experiments due to the time of measurements (1:00 P.M. to 2:00 P.M.). Similar observations of atmospheric conditions at the same experimental site are noticed by (Khallaf et al. 2021). With a fluid flow rate of 0.03 Kg/s both thermal and electrical properties have been measured as depicted in Fig. 7(a–f). Figure 7 (a) and (b) offer the values of the short-circuit current (*I*_*sc*_) and the open-circuit voltage (*V*_*oc*_) of the three panels when measured for a quarter-hour from the cooling starting time. It has been shown that the *I*_*sc*_ resulting from the panel cooled by nanofluid is higher than the reference *I*_*sc*_ by 0.2 Amp., whereas the difference has increased by 0.8 volts for the *V*_*oc*_*.* For the water-cooled panel, the *I*_*sc*_ is lower than the third panel by 0.18 Amp. Based on the results obtained from Fig. 7(d), the overall electrical efficiencies of the three panels have been calculated. The third panel has the highest electrical efficiency in comparison to the second and first ones. The efficiencies of the three PV panels are 12.94%, 12.53%, and 11.99% for the third, second, and first panels, respectively.

**Fig. 8:**
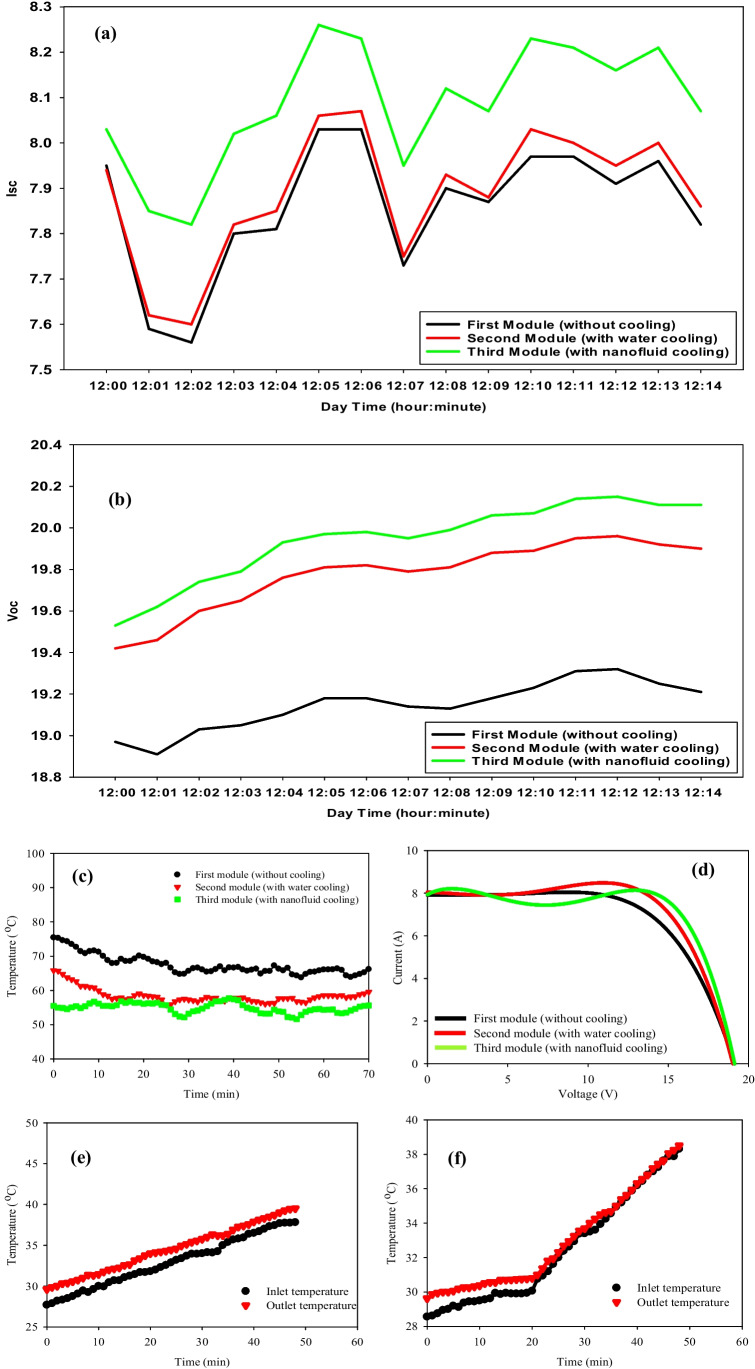
Electrical properties and temperature of the PV modules due to a mass flow rate of 0.07 kg/s and concentration of 0.05% (26–08-2021). (**a**) Short-circuit current vs. time of the three PV modules (12:00 P.M. to 12:15 P.M.). (**b**) Open-circuit of the three PV modules (12:00 P.M. to 12:15 P.M.). (**c**) Modules surface temperature vs. time (12:00 P.M. to 1:10 P.M.). (**d**)* I*–*V* characteristics of the three PV modules. (**e**), (**f**) Inlet and outlet temperatures of the 2nd and 3rd PV modules vs. time (12:00 P.M. to 12:50 P.M.)

To understand the relationship between the temperature of the PV panel and *I*_*sc*_/*V*_*oc*_, the experiments are carried out without a cooling medium (reference panel). The average PV panel surface temperature of each one is measured every minute, presented in Fig. 7(c). The results show that the temperature of the reference PV panel varies from 52.5 to 70.5 °C with an average panel temperature of 62.5 °C. Also, the average PV panel temperature cooled by water and by nanofluid is observed to be about 58 and 54 $^\circ{\rm C}$, respectively. On the other hand, the inlet and outlet of both fluids—water and nanofluid—are shown in Fig. 7(e) and (f). Nanofluid’s temperature behavior is not as steady as water. In Fig. 7(c), the second panel which has a serpentine filled with water is initially the highest one in temperature, then plunged through the first 20 min, in contrast to the third panel given the lowest values of temperatures during the time of measurements (1 h). The steady decrease in the three curves is because of solar irradiance decrement as a function of time which increases after reaching the maximum value of solar radiation. Figure 8 (a–f) shows the electrical properties and temperature distributions of the three PV modules with a flow rate of 0.07 kg/s. The flow rate of 0.07 kg/s has shown better values of *I*_*sc*_/*V*_*oc*_ and overall efficiency than the latter, as shown in Fig. 8(a), (b), and (d) for the same concentration (i.e., 0.05%). These data are taken on the 26th of August with an average of solar radiation of 960 W/m^2^ and ambient air temperature of 36.4 °C observed from 12:00 P.M. to 1:00 P.M. The average improvement in the short-current circuit is 0.25 Amp. for nanofluid cooling and 0.02 Amp. for water cooling, while the *V*_*oc*_ is improved by 0.85 and 0.65 V for the same techniques over the reference. The overall efficiencies are 12.156%, 12.666%, and 13.419% for the reference module, water cooling module, and nanofluid cooling module, respectively as shown in Fig. 8(d). As known, the rise in panel temperature negatively affected the open-circuit voltage, and that in turn reduced the electrical efficiency of the PVT system. This is due to the change of resistance in the panels as the temperature increases which results in a drop in voltage in the electrical circuit. An agreement with our results is reported by Aste et al. (2016) for obtaining lower PV efficiency at higher panel temperatures because of a drop in voltage for the uncooled PVT system. To improve the electrical power output of PV modules and avoid overheating, water and Al_2_O_3_-based nanofluid are used as cooling media. With nanofluid cooling, the open-circuit voltage increased by 5 to 6% compared to an uncooled PV panel system as the panel surface operating temperature is significantly reduced, also, an improvement of the fill factor parameter by 3.05% with nanofluid cooling in comparison to the uncooled PV system (0.05% concentration and flow rate of 0.07 kg/s). Figure 8 (c) demonstrates that the temperature of the third panel is the lowest value, which leads to higher energy output than the other two panels. The maximum reduction of temperature is 20 °C (i.e., 35.7%), while it is 8 °C (i.e., 14.1%) at the lowest value of reducing temperature as for nanofluid cooling, in comparison to 11 °C (i.e., 19.6%) and 8 °C for water cooling. The increment of both curves shown in Fig. 8(e) and (f) is due to the gradual increase of the inlet temperature of the fluids, as a closed system with no external sources of cooling. From Fig. 8(e) and (f), it is observed that at lower flow rates, the outlet water temperature is high but as the flow rate increases, the outlet temperature reduces. Also, it is noticed that the rise in temperature decreases with an increase in mass flow rate. The effect of radiation intensity on the temperature rises shows that the temperature rise increases with an increase in radiation intensity at same the flow rate (Menon et al. 2022; Bahaidarah et al. 2013; De Soto et al. 2006).

### With fluid concentration = 0.03%

**Fig. 9:**
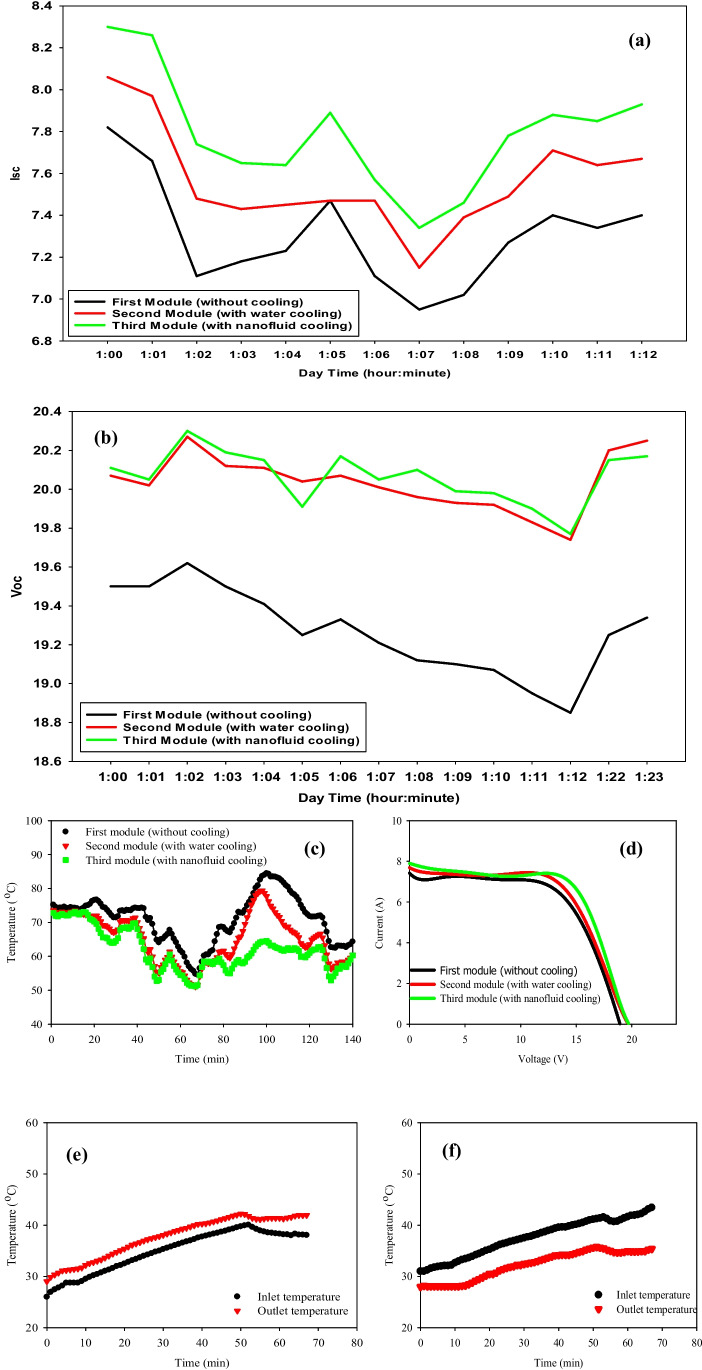
Electrical and temperature distribution properties of PV panels due to mass a flow rate of 0.03 kg/s [14–09-2021]. (**a**) *I*_*sc*_ vs. time of the three PV modules (1:00 P.M. to 1:12 P.M.). (**b**) *V*_*oc*_ vs. time of the three PV modules (1:00 P.M. to 1:12 P.M.). (**c**) Module surface temperature vs. time (12:30 P.M. to 2:00 P.M.). (**d**) *I*–*V* characteristics of the three PV modules. (**e**), (**f**) Inlet and outlet temperatures of the 2nd and 3rd PV modules vs. time (12:54 P.M. to 2:00 P.M.)

On September 14, 2021, the average calculated solar radiation is 910 W/m^2^ during the period of data measured. The ambient temperature starting point is 32.63 °C reaching 42.56 °C at 14:00 P.M. As shown in Fig. 9(a), the highest values of *I*_*sc*_ are 8.3, 8.03, and 7.82 Amp., and for the *V*_*oc*_ are 20.3, 20.25, and 19.62 V for nanofluid, water cooling, and uncooling PV panels, respectively, with a flow rate of 0.03 kg/s. Figures 7 (d) and 9 (d) have been carried out at the same mass flow rate (0.03 kg/s), but under different values of solar radiation, an enhancement in the overall efficiency by 10.66% has been obtained. Figure 9 (c) demonstrates that the behavior of panels’ surface temperature as a function of time is illustrated from 12:30 P.M. to 12:54 P.M. which is the time of starting the cooling process. As it is clear, the second and third PV panels’ surface temperatures are asymptotic during this period, while the difference will be obvious until 1:47 P.M. This is the time, by which the pumps are shut down to observe how long it takes for the second and third panels’ surface temperatures will be equal. However, it is shown that the existence of fluid under the panels works as a heat absorber for a long time’ nevertheless, the forcing is needed for better enhancement. It is seen from Fig. 9(c), that nanofluid cooling of the PV panel resulted in a significant reduction in the panel temperature, especially from 12:57 P.M. to 13:51 P.M. The panel temperature varied from 52.5 to 63.75 °C with an average panel temperature of 58.125 °C, while the average panel temperature during the same time is 69.5 and 74.25 °C for water-cooled and uncooled PVT systems, respectively. The average panel surface temperature reduction of 16.125, °C 21.71% and 11.375 °C, 15.15% is obtained for the nanofluid-cooled PVT system over the uncooled and water-cooled PVT system, respectively. Figure 9 (e) and (f) show the obtained results of the inlet and outlet temperatures of the water-cooled and nanofluid PVT panels have been recorded from 12:54 P.M. to 1:47 P.M.; this is the period of pumping the fluid, while after that time, the inlet and outlet temperatures are just for stationary fluids. Figure 9 (e) and (f) show hourly variations in the inlet and outlet temperatures of water and nanofluid in the PVT system. The temperature of water at the inlet and outlet of the PVT system varied from 29.5 to 39.5 °C and 31.5 to 43.5 °C, respectively. While that for nanofluid are 28. to 35 $^\circ{\rm C}$ and 31 to 41.0 $^\circ{\rm C}$ for inlet and outlet temperatures, respectively. The temperature rise, i.e., (*T*_outlet_–*T*_inlet_) for both nanofluid-cooled and water-cooled was nearly the same. The calculated overall electrical efficiency of nanofluid and water over the reference PV panel are 14.64% and 5.49%, respectively.

**Fig. 10:**
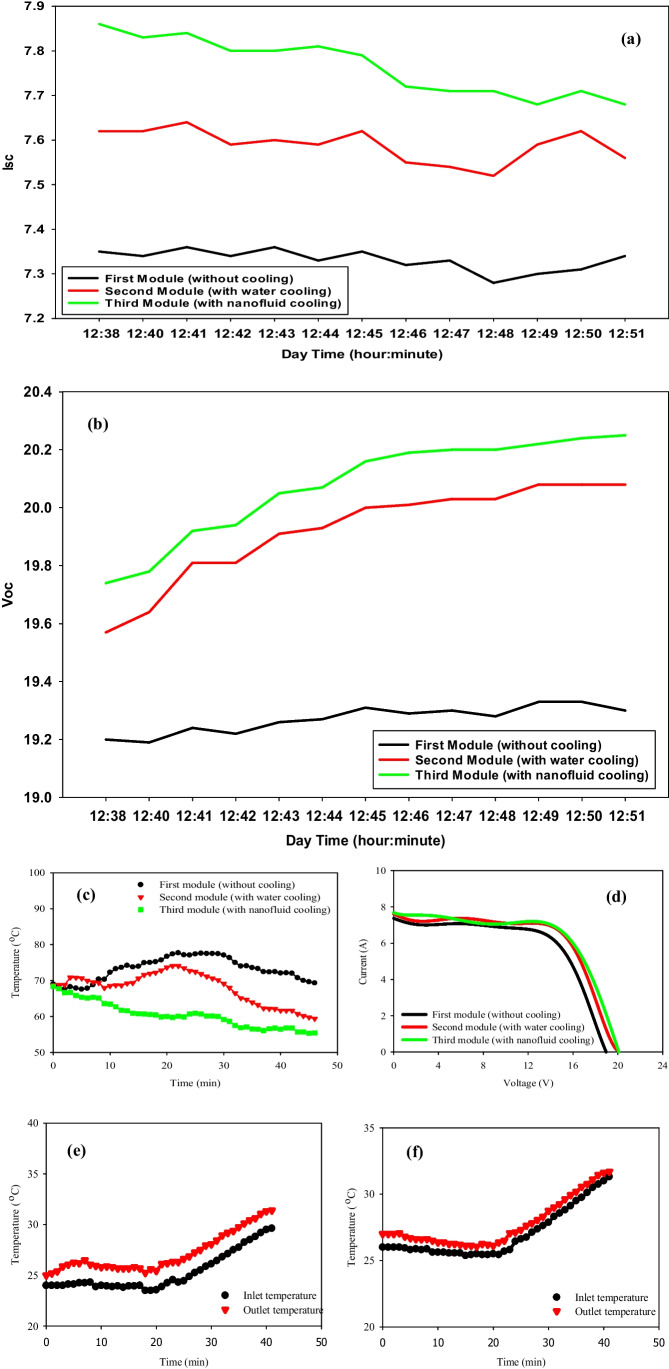
PV properties due to the mass flow rate of 0.07 kg/s [12–09-2021]. (**a**) *I*_*sc*_ vs. time of the three PV modules (12:38 P.M. to 12:51 P.M.). (**b**) *V*_*oc*_ vs. time of the three PV modules (12:38 P.M. to 12:51 P.M.). (**c**) Modules surface temperature vs. time (12:33 P.M. to 1:20 P.M.). (**d**) *I*–*V* characteristics of the three PV modules. (**e**), (**f**) Inlet and outlet temperatures of the 2nd and 3rd PV modules vs. time (12:38 P.M. to 1:20 P.M.)

On the 12th of September 2021, another run throughout a time interval from 12:30 P.M. to 1:30 P.M. has been done with a mass flow rate of 0.07 kg/s. The average measured solar radiation is 909 W/m^2^, and the starting ambient temperature is 28.06 °C reaching 29.12 °C at the end of the measurement, which reflects the slight steady growth of temperature. The nanofluid cooling and water cooling record higher differences in the values of *I*_*sc*_ and *V*_*oc*_ with an average of 0.4, 0.27 Amp. and 0.85, 0.61 V yield of the nanofluid-cooled and water-cooled panels over the reference PV panel presented in Fig. 10(a) and (b). Based on the results in Fig. 12b, the calculated overall efficiency is 11.836%, 12.994%, and 13.346% for the reference, second, and third PV panels; whereas, it reaches 12.71% and 9.74% of improvement for nanofluid-cooled and water-cooled panels in contrast with the reference PV panel. Figure 10 (c) shows the hourly average surface temperature of the three PV panels throughout the time of the experiment (12:33 P.M. to 1:30 P.M.). As shown in the figure, the surface temperature of the nanofluid PV panel is 60 °C, which is the lowest among the other two panels, followed by the water-cooled 69.5 $^\circ{\rm C}$ and 75.25 $^\circ{\rm C}$ for the reference PV panels, respectively. This is due to a drop in the average temperature absorbed by the nanofluid and water-cooled at the backside iron serpentine of the PV systems. On the other hand, surface temperature for nanofluid cooling, water cooling, and reference PV systems has the same pattern behavior during the experiment. Figure 10 (e) and (f) represent the behavior of the inlet and outlet temperatures of both water-cooled and nanofluid-cooled PV panels. The time of the experiment is from 12:40 P.M. to 1:20 P.M. Both inlet and outlet temperatures of both water and nanofluid colling have the same behavior trend. But the behavior can be divided into two parts: (i) from 12:40 P.M. to 1:01 P.M. for both water and nanofluid, a constant pattern has been obtained, and (ii) from 1:01 P.M. to 1:20 P.M., a linear incremental relationship has been obtained. Also, the temperature rise (*T*_outlet_ – *T*_inlet_) has been reduced with nanofluid 1 °C in comparison to 3 °C for water cooling.

### With nanofluid concentration = 0.01%

On the 21st of September 2021, the average calculated value of the solar radiation is 683 W/m^2^ and the mean ambient air temperature is 31.5 °C throughout the experiment (the graphs of the data are not included). From the results, the value of the solar radiation 683 W/m^2^ is the lowest one in comparison to the values obtained on the other days of September.

**Fig. 11:**
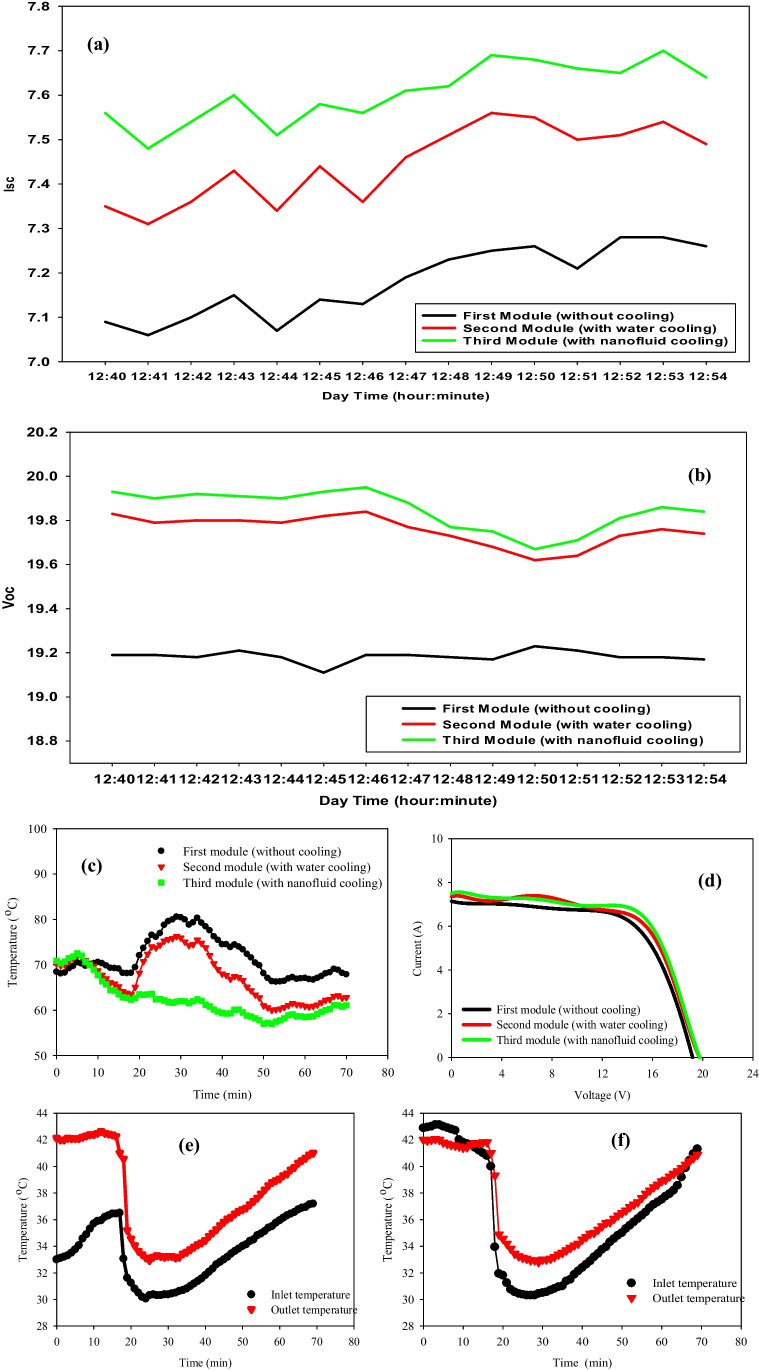
PV modules properties due to the mass flow rate of 0.03 kg/s [21–09-2021]. (**a**) *I*_*sc*_ vs. time of the three PV modules (12:40 P.M. to 12:54 P.M.). (**b**) *I*_*sc*_ vs. time of the three PV modules (12:40 P.M. to 12:54 P.M.). (**c**) Module surface temperature vs. time (12:35 P.M. to 1:52 P.M.). (**d**) I–V characteristics of the three PV modules. (**e**), (**f**) Inlet and outlet temperatures of the 2nd and 3rd PV modules vs. time (12:35 P.M. to 1:25 P.M.)

The average increment *I*_*sc*_ and *V*_*oc*_ yield for the third panel cooled by nanofluid are 0.43 Amp. and 0.71 volts, while it is 0.27 Amp. and 0.57 volt for the panel cooled by water over the first one (reference). Moreover, from 12:40 P.M. to 12:54 P.M., *I*_*sc*_ and *V*_*oc*_ of the nanofluid-cooled PVT system with a nanofluid concentration of 0.01% and flow rate of 0.03 Kg/s were always better than that with water-cooled and uncooled PV panel system, as depicted in Fig. 11(a) and (b). The short-current circuit and open-circuit voltage yield of a PVT panel cooled by nanofluid is better than that of cooled by pure water (base fluid) for all cases of flow rates because the back-sheet temperature of PVT with nanofluid is lower than that of both water-cooled PVT panel and uncooled one. The electrical instantaneous power increases by increasing the rate of the following fluid, due to the cooling effect that reduces the temperature of the back sheet.

The electrical efficiency of a PV system decreases by increasing the temperature. According to the silicon absorbance capability, most of the incident solar energy is converted to electrical energy, while the remaining is converted to heat energy inside the photovoltaic cells. Increasing the flow rates of nanofluid increases electrical efficiency. Increasing the volumetric concentration of Al_2_O_3_/water nanofluid improves electrical efficiency due to increased heat transfer rate. Based on the data obtained in Fig. 11(d), the overall calculated efficiency can be calculated. The overall efficiency is 13.188%, 13.528%, and 13.807% for reference, water-cooled, and nanofluid-cooled PV panels, respectively. Hence, the enhancement of nanofluid cooling is 4.7 and 2.6% for water cooling. The impact of nanofluid cooling and water cooling on the temperature (front surface) of the PVT panels throughout the time from 12:35 P.M. to 1:52 P.M. is shown in Fig. 11(c). The cooling process started at 12:25 P.M. The increase in temperature until 12:56 P.M. is due to higher solar radiation values during this interval. The measurement process ended at 1:40 P.M.; at this moment, it takes 5 min for the second and third panels to be equal in temperature. The variation of cell temperature for nanofluid cooling, water cooling, and the non-cooling cases is presented, and the average PV panel temperatures are 60.5 °C, 68.0 °C, and 72.5 °C, respectively. Nanofluid cooling of the PV panel resulted in a reduction of the cell temperature by 16.55% over the uncooled one, while the water-cooled PVT panel has a reduction percentage of 6.2% over the uncooled one. At the same time, from 12:39 P.M. to 1:13 P.M., the nanofluid cooling PVT system has its maximum surface temperature reduction of 25 °C over the uncooled one. In addition, as indicated, all PV and PVT systems have a temperature above the ambient temperature. This means that the active nanofluid PVT’s cooling mechanism is more efficient than water cooling. The inlet and outlet temperatures of the nanofluid and water are given in Fig. 11(e) and (f). From the results, during the time interval from 12:35 P.M. to 12:55 P.M., we started the cooling process, so it is characterized by its high temperature with a sharp drop edge in temperature at 12:55 P.M. After that to the end of the experiment, both inlet and outlet temperatures of both water and nanofluid cooling have normal behavior. i.e., gradually increase with time.

**Fig. 12:**
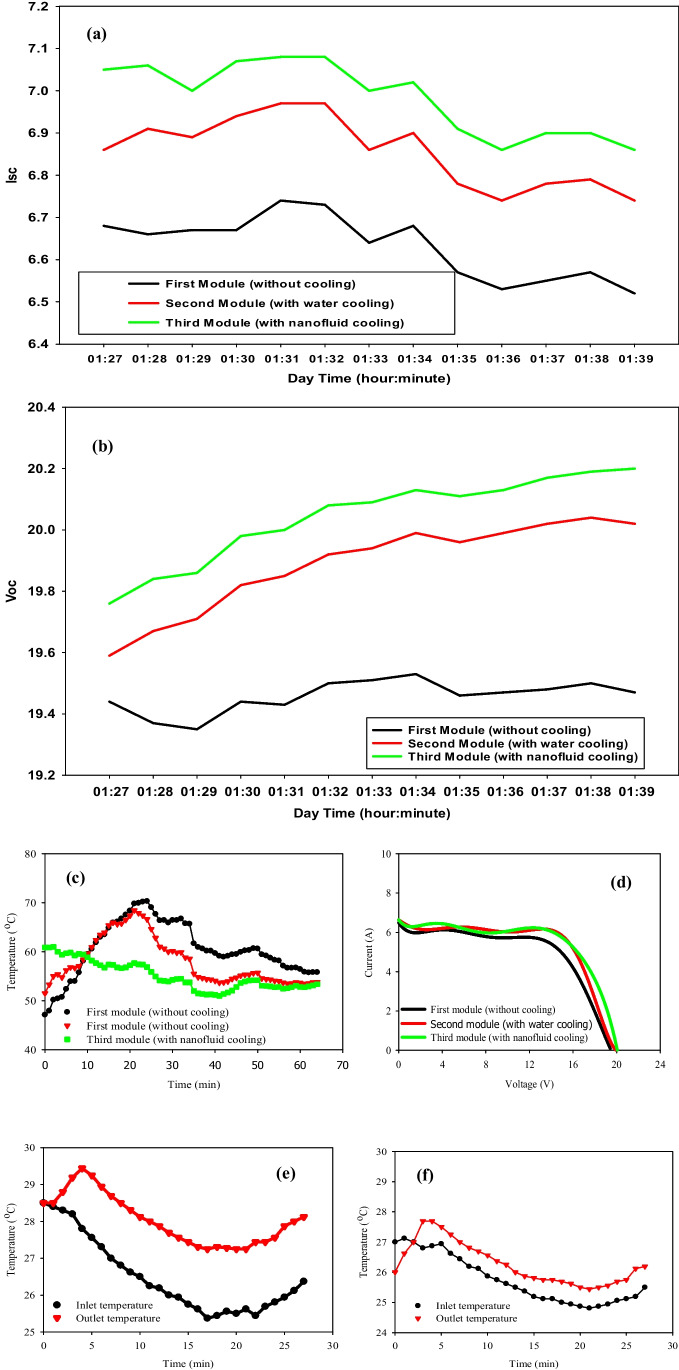
PV properties due to the mass flow rate of 0.07 kg/s [19–09-2021]. (**a**) *I*_*sc*_ vs. time of the three PV modules (1:27 P.M. to 1:39 P.M.). (**b**) *V*_*oc*_ vs. time of the three PV modules (1:27 P.M. to 1:39 P.M.). (**c**) module surface temperature vs. time (1:24 P.M. to 2:24 P.M.). (**d**) I–V characteristics of the three PV modules. (**e**), (**f**) Inlet and outlet temperatures of the 2nd and 3rd PVT modules vs. time (1:27 P.M. to 1:53 P.M.)

Figure 12 (a–f) represents the electrical performance and temperature distribution of the three PV panels on September 19, 2021. The time interval of measurements ranged from 1:27 P.M. to 1:56 P.M. The calculated hourly mean solar radiation at the period of measurement is 683 W/m^2^ with an average ambient temperature of 35 °C. Figure 12 (a) explains that the average increment in *I*_*sc*_ and *V*_*oc*_ for the third panel is 0.35 Amp. and 0.63 volts, while it is 0.23 Amp. and 0.45 volts for the panel cooled by water over the reference PV panel. It is strongly dependent on the solar radiation values and the module temperature. Based on the results in Fig. 12(d), the overall efficiency can be calculated. The estimated values of the overall efficiency are 12.848%, 13.929%, and 14.8399% for the first, second, and third panels. Hence, the enhancement of nanofluid cooling is by 15.5%, and by 8.41% for water cooling. The cooling process started at 1:27 P.M., and as it is clear in Fig. 12(c), the temperature of the first 7 minutes of the second and third panels is higher than the first panel. In contrast, they started to go down at 1:29 P.M., after 2 minutes of cooling. Then, the third panel’s temperatures continue to be the lowest. The sudden increment in inlet and outlet temperatures at 1:55 P.M. for both, the third and second modules is due to stopping the cooling process. Furthermore, the presence of Al_2_O_3_ nanoparticles boosts the thermal conductivity of the base fluid, which improves the cooling process and results in a good reduction in the surface PV panel temperature.

Figure 12 (e) and (f) demonstrate the inlet and outlet temperatures of both water-cooled and nanofluid-cooled PV panel systems with a mass flow rate of 0.07 kg/s the temperature rise ($${T}_{outlet}-{T}_{inlet})$$ with nanofluid cooling is decreased than that for water cooling PV system.

Finally, a comprehensive comparison of the impact of nanofluid cooling on the maximum power (*P*_*max*_) at the maximum power point, short-circuit current (*I*_*sc*_) and open-circuit voltage (*V*_*oc*_) yield, and the surface operating temperature of the PV panel system under investigation are summarized in Tables 5, 6, and 7. From these tables, it is observed that (i) both P.M. instantaneous output power yield are strongly dependent on the nanofluid concentration and mass flow rate, (ii) a reduction of 22.88% in surface operating temperature of the PV panel (with 0.05% concentration and mass flow rate 0.07 kg/s) in comparison to uncooling PV one has been obtained.

**Table 5:** Maximum power of the PVT panel systems for uncooling, water cooling, and nanofluid cooling

Concentration (%)	Mass flow rate (Kg/s)	${P}_{m}$(W)		
		Uncooling	Water cooling	Nanofluid cooling
0.01	0.03	87.50	95.14	99.73
	0.07	76.42	75.15	88.47
0.03	0.03	84.65	93.16	100.11
	0.07	89.85	96.78	98.20
0.05	0.03	85.25	90.77	91.34
	0.07	98.16	101.22	108.40

**Table 6:** Average *I*_*sc*_ and *V*_*oc*_ yield of PVT panel systems for water cooling and nanofluid cooling over the uncooled panel

Concentration (%)	Mass flow rate (Kg/s)	Average *I*_*sc*_ and *V*_*oc*_ yield over the reference	
		Water cooling (*I*_*sc*_) (*V*_*oc*_)	Nanofluid cooling (*I*_*sc*_) (*V*_*oc*_)
0.01	0.03	0.27 0.57	0.43 0.71
	0.07	0.23 0.45	0.35 0.65
0.03	0.03	0.28 0.80	0.50 0.82
	0.07	0.27 0.61	0.40 0.85
0.05	0.03	0.04 0.68	0.20 0.80
	0.07	0.02 0.85	0.25 0.65

**Table 7:** Surface operating temperature of the PVT panel systems for uncooling, water cooling, and nanofluid cooling

Concentration (%)	Mass flow rate (Kg/s)	Solar panel’s temperature (°C)		
		Uncooling	Water cooling	Nanofluid cooling
0.01	0.03	69.90	66.32	63.09
	0.07	60.58	57.96	55.11
0.03	0.03	70.94	65.32	61.78
	0.07	73.14	67.97	60.39
0.05	0.03	62.00	61.76	55.36
	0.07	67.46	58.42	54.92

## Error and uncertainty of the experiment

In the experimental work, there is a possibility of obtaining a non-accurate result during the measuring process. Hence, the uncertainty of measuring should be clarified and taken into consideration. In this work, we depend on the calculation of the uncertainty in the model presented by (Hasani and Rahbar 2015). The accuracy and uncertainty ranges are presented in Table 8, using the following equation:

**Table 8:** The uncertainties during the various parameters measuring

Instrument	Accuracy (*a*)	Range	Standard uncertainty (*u*)
Ammeter	± (0.8% + 8) Amp.	0-20 Amp.	0.046 Amp.
Voltmeter	± (0.5% + 2) Volt	0–1000 Volt	0.028 Volt
Temperature sensor	± 0.5 °C	− 55 − + 125 °C	0.29 °C
Flowmeter	± 2%	0 – 60 L/min.	0.01 L/min.
Pyranometer	± 1.0 W/m^2^	0–1200 W/m^2^	0.58 W/m^2^

$$u=\frac{a}{\sqrt{3}}$$

As (*u*) is the standard uncertainty and (*a*) is the accuracy of the measuring tools.

## Conclusions

In this study, a photovoltaic thermal (PVT) system with a serpentine coil-configured sheet and plate thermal absorber setup is fabricated, and then electrical and temperature distribution performance is evaluated using water and nanofluid. The results revealed that the cooling of PVT panels with water and nanofluid can substantially improve the electrical and surface temperature performance of the system. The system is tested under climate conditions in Tanta city with a ${29.25}^{^\circ }$ latitude angle), Egypt. Based on the obtained results, the following conclusions are drawn:

i- The alumina Al_2_O_3_ nanofluid, as expected, has shown better improvement in the electrical characteristics of the panel over water and the reference module.
ii- For the same nanoparticles concentration, it was found that the enhancement is getting lowered as the mass flow rate is decreased, and the same argument for water cooling.
iii- The PV module conversion efficiency is sensitive to its surface operating temperature and decreases as the PV temperature increases.
iv- With active nanofluid cooling, the surface operating temperature of the PV module dropped significantly to about 22.83%.
v- The open-circuit voltage (*V*_*oc*_) and the short-circuit current (*I*_*sc*_) are measured in the first 15–20 min of the cooling process.
vi- Finally, the temperature rise (*T*_outlet_–*T*_inlet_) of the nanofluid as a function of flow rate is studied. With an increase in the mass flow rate of the nanofluid, the temperature rise reduced.
vii- For future research work, we recommend the usage of more thermally conductive nanofluids. It would be better to narrow the spaces between the pipes in the back of the module, which will cover more area to be cooled, hence, improving the cooling process and efficiency.

## Data Availability

The data presented in this study and materials used are available in the context of the article.
